# Early Childhood Caries: Prevalence, Risk Factors, and Prevention

**DOI:** 10.3389/fped.2017.00157

**Published:** 2017-07-18

**Authors:** Sukumaran Anil, Pradeep S. Anand

**Affiliations:** ^1^Department of Preventive Dental Sciences, College of Dentistry, Prince Sattam Bin Abdulaziz University, AI-Kharj, Saudi Arabia; ^2^Department of Dentistry, ESIC Medical College, Parippally, India

**Keywords:** dental caries, early childhood caries, dietary habits, oral health, pediatric oral health, sociodemographic factors, infant feeding

## Abstract

Early childhood caries (ECC) is major oral health problem, mainly in socially disadvantaged populations. ECC affects infants and preschool children worldwide. The prevalence of ECC differs according to the group examined, and a prevalence of up to 85% has been reported for disadvantaged groups. ECC is the presence of one or more decayed, missing, or filled primary teeth in children aged 71 months (5 years) or younger. It begins with white-spot lesions in the upper primary incisors along the margin of the gingiva. If the disease continues, caries can progress, leading to complete destruction of the crown. The main risk factors in the development of ECC can be categorized as microbiological, dietary, and environmental risk factors. Even though it is largely a preventable condition, ECC remains one of the most common childhood diseases. The major contributing factors for the for the high prevalence of ECC are improper feeding practices, familial socioeconomic background, lack of parental education, and lack of access to dental care. Oral health plays an important role in children to maintain the oral functions and is required for eating, speech development, and a positive self-image. The review will focus on the prevalence, risk factors, and preventive strategies and the management of ECC.

## Introduction

Early childhood caries (ECC) has been on the increase in many countries and has become a significant health problem especially in socially disadvantaged populations. ECC is defined as the presence of one or more decayed, missing, or filled tooth surfaces in any primary tooth in a child at 71 months of age or younger. It has several unique characteristics in clinical appearance such as rapid development of caries, which affects a number of teeth soon after they emerge in oral cavity. These lesions involve tooth surfaces that are less prone to caries development. Several terminologies were used to describe the condition such as, nursing bottle caries, nursing caries, rampant caries, baby bottle caries, baby bottle tooth decay, milk bottle syndrome, and prolonged nursing habit caries. ECC is a multifactorial disease that results from the interaction of factors that include cariogenic microorganisms, exposure to fermentable carbohydrates through inappropriate feeding practices, and a range of social variables. ECC is a severe health condition found among children living in socially disadvantaged communities in which malnutrition is a social and health disparity (1, 2). ECC is associated with other health problems, ranging from local pain, infections, abscesses, leading to difficulty in chewing, malnutrition, gastrointestinal disorders, and difficulty in sleeping (3).

**Figure 1:**
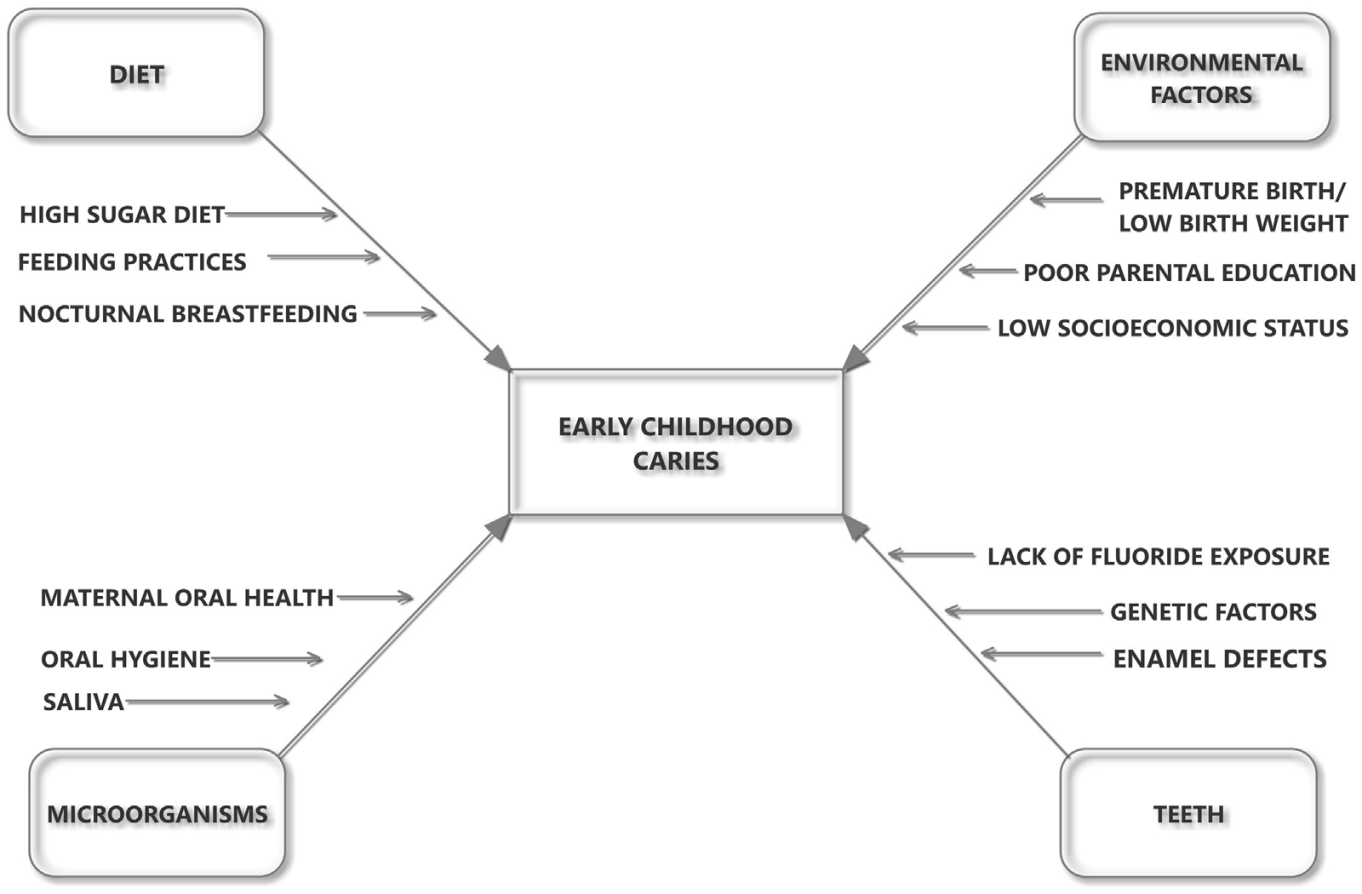
The influence of host–microbe–diet interactions in the etiology and pathogenesis of early childhood caries.

The etiology of ECC is multifactorial and is mainly attributed to a time-specific interaction of microorganisms with sugars on a tooth surface (Figure 1) (4). Diet and feeding practices also play an important role in acquisition of the infection and development of caries (5, 6). Factors such as high sugar intake, lack of oral hygiene, lack of fluoride exposure, and enamel defects are some of the major factors responsible for the development of ECC (7–9). ECC is higher among the more socially disadvantaged and particularly for children who are refugees or migrants, or whose parents are refugees or migrants from third world countries (10, 11). This could be related to low socioeconomic status, social exclusion, and sociocultural differences in oral health beliefs and practices (12). ECC is a serious oral health problem, especially in disadvantaged communities in both developing and industrialized countries in which undernutrition is very common (13).

## Epidemiology of ECC

Despite the decline in the prevalence of dental caries in children in western countries, caries in preschool children remains a major problem in both developed and developing countries (13). Prevalence of ECC also varies widely with several factors like race, culture, and ethnicity; socioeconomic status, life style, dietary pattern, and oral hygiene practices and also according to the various factors from country to country and from area to area. A review of the literature suggests that in most developed countries the prevalence rate of ECC is between 1 and 12% (14). In less developed countries and among the disadvantaged groups in the developed countries, the prevalence has been reported to be as high as 70%. ECC has been found to be more prevalent in low socioeconomic groups (15, 16). The prevalence ranged from 11.4% in Sweden to 7–19.0% in Italy (17, 18). A high prevalence of ECC has been reported in some Middle Eastern countries, such as Palestine (76%) and the United Arab Emirates (83%) (19, 20). The national surveys from some countries, such as Greece (36%), Brazil (45.8%), India (51.9%), and Israel (64.7%), showed inconsistent prevalence of ECC (21–24). In a systematic review, Ismail and Sohn (25) found that the prevalence varied from 2.1% in Sweden to 85.5% in rural Chinese children. The national prevalence of ECC in the USA can be estimated between 3 and 6%, which is consistent with the prevalence in other western countries (26, 27). According to a study, the highest prevalence of ECC is found in the 3- to 4-year-old age group and that boys are significantly more affected than girls, aged between 8 months and 7 years (28). Epidemiological studies from Europe showed significant percentages of the preschool children are affected by ECC, confirming the widespread prevalence of the disease. ECC is randomly dispersed in the population, with the disease affecting disproportionately among deprived families (29).

## Etiology of ECC

Dental caries results from the interaction of various etiological factors, which might be concurrently present to initiate and progress the disease. The factors are (1) cariogenic microorganisms, (2) fermentable carbohydrates (substrate), and (3) susceptible tooth surface/host. There are a multitude of risk factors associated with ECC. Epidemiological studies have also documented low socioeconomic status, minority status, low birth weight, and transfer of microbes from mother to child. One to twelve percent of children younger than 6 years in the developed world experience ECC (30, 31). Child oral health-care behavior, feeding and cleaning behavior are associated with ECC among children; night time bottle feeding and frequent consumption of cariogenic food; late commencement of child tooth brushing and irregular brushing habits (32–34).

### Cariogenic Microorganisms

*Streptococcus mutans* (SM) and *Streptococcus sobrinus* are the most common microorganisms associated with ECC. Lactobacilli also participate in the development of caries lesions and play an important role in lesion progression, but not its initiation (35). SM metabolizes sugars to produce acids, which contribute to the demineralization of tooth structure (36). These bacteria can be transmitted from mother to child (37). Preschool children with high levels of SM in the oral cavity had higher caries prevalence and a greater risk for development of new lesions (38). Milgrom et al. (9) found that children having a high SM levels were five times more prone to have dental caries. The major source of acquiring the SM is from the mother during first 12–24 months. Poor maternal oral hygiene maintenance and frequent snacking and sugar exposure increase the chances of transmission of the infection to child (39). SM isolates from infants indicated that the source of the SM in children is mainly from their mothers *via* vertical transmission through saliva (40, 41). Horizontal transmission of microbes may occur between siblings and care givers (42, 43). Infants delivered by cesarean section acquire SM earlier than vaginally delivered infants since these deliveries are more aseptic and the atypical microbial environment increases the chances of SM colonization (44). The *Actinomyces* species and specifically *Actinomyces gerencseriae* were also associated with caries initiation (45), while *Bifidobacterium* species was associated with deep caries lesions (46). Few non-mutans streptococci that have acidogenic and aciduric properties were also associated with caries (47, 48). Epidemiological data suggest that *Candida albicans* also plays an active role in the pathogenesis of dental caries (49, 50). SM is the main bacteria that have strong association to dental caries whereas the other oral bacteria in the dental plaque could be involved in the initiation and progression of caries.

### Diet

Dietary practices also play a significant role in the development of ECC especially if it contains high levels of fermentable carbohydrates; the child is at higher risk for dental caries (51). Inappropriate feeding practice can prolong the exposure of teeth to fermentable carbohydrates which in turn may aggravate the chances of ECC. Bottle feeding during bedtime or sleeping has been associated with the initiation and development of caries in children (52). SM converts fermentable carbohydrates into acids, which can demineralize enamel and dentin (53). Studies have shown that cow milk has minimal cariogenicity due to its mineral content and low lactose level (54–56). Iida et al. (57) showed that breast feeding and its duration were independently associated with an increased risk for ECC among 2- to 5-year-old children. A systematic review revealed that breast feeding for more than a year and at night might be associated with an increased prevalence of dental caries (58). Infant feeding practices such as frequent exposure to sugar, frequent snacking, taking sweetened drinks to bed, sharing foods with adults, as well as maternal caries status, oral hygiene and dietary habits predispose to early SM colonization and establishment of high MS counts (51).

### Environmental Factors

Lack of good oral hygiene practices promotes the development of ECC. Children should begin receiving oral hygiene care upon the eruption of the first primary tooth (59). Children from low socioeconomic status are two times more likely to have dental caries than from higher income strata (60). Caregivers’ social status, poverty, ethnicity, deprivation, number of years of education, and dental insurance coverage are other factors which influence the oral hygiene habits of children and the severity of ECC (28, 61).

Saliva has a protective role against dental caries development by providing the main defense system. Saliva flow rate, antimicrobial properties, the buffering capacity, and clearance of foods from the oral cavity are factors that are important in reducing the development of caries (62). Feeding of high sugar containing food at night may increase the caries risk for infants and toddlers due to the low salivary flow rate (63). Studies have shown the presence of enamel hypoplastic defects with prenatal conditions such as premature birth and low birth weight, as well as with malnutrition and illness (64, 65). In a case–control study, enamel hypoplasia was reported in 67% of low birth weight children in comparison to 10% among normal birth weight children (66). Although enamel hypoplasia has been confirmed as an independent risk factor for caries, the causal relationship with dental caries has not been established. Low socioeconomic status, poor parental education, and life style factors have significant influence on ECC (67). Leroy et al. (68) reported a significant relationship between parental smoking habit and caries experience children.

## Clinical Presentation

ECC is a form of early, moderate and late dental decay that affects the primary teeth of infants and toddlers. It develops on tooth surfaces that are usually at low risk for caries, such as the labial surfaces of maxillary incisors and lingual and buccal surfaces of maxillary and mandibular molars. ECC initially presents as dull white or brown spots on maxillary incisors along the gingival margin, which progresses to a complete destruction of the crown, leading to root stumps (1). In moderate stage, the caries begins to spread to the maxillary molars. In the severe stage, the caries process destroys the maxillary teeth and spreads to the mandibular molars. Based on the clinical appearance, attempts were made to classify the ECC (27). Severe early childhood caries (S-ECC) refers to children with ‘atypical,’ ‘progressive,’ ‘acute,’ or ‘rampant’ pattern of dental caries (25, 69). A child with ECC may suffer from considerable pain, which may lead to difficulty in eating and talking (70). If the extent of the damage results in extraction of the anterior teeth by age 2 or 3 years, the child may suffer further developmental delays involving speech articulation and patterns (71). The consequences are delay in physical development due to poor nutrition and the pain and discomfort may compromise their desire to eat. The pain and suffering associated with the caries affect the child’s oral health quality of life (72).

## Classification

Several research groups have attempted to develop classification systems for early childhood caries (Tables 1–3).

**Table 1:** Classification based on the severity of ECC and etiology (73).

Type I (mild to moderate)	The existence of ‘isolated carious lesion(s)’ involving incisors and/or molars. The most common causes are usually a combination of semisolid or solid food and lack of oral hygiene.
Type II (moderate to severe)	ECC was described as ‘labiolingual lesions’ affecting maxillary incisors, with or without molar caries, depending on the age of the child and stage of the disease. Typically, the mandibular incisors are unaffected. The cause is usually inappropriate use of a feeding bottle or at-will breast-feeding or a combination of both, with or without poor oral hygiene.
Type III (severe)	ECC was described as carious lesions affecting almost all teeth including the mandibular incisors. A combination of cariogenic food substances and poor oral hygiene is the cause of this type of ECC.

**Table 2:** Classification based on the pattern of ECC presentation (27).

Type 1	Lesions associated with developmental defects (pit and fissure defects and hypoplasia)
Type 2	Smooth surface lesions (labial-lingual lesions, approximal molar lesions)
Type 3	Rampant caries—having caries in 14 out of 20 primary teeth, including at least one mandibular incisor

**Table 3:** Classification of ECC and Severe Early Childhood Caries (S-ECC) (1, 69).

Age (months)	Early childhood caries	Severe early childhood caries
<12	1 or more dmfs surfaces	1 or more smooth dmf surfaces.
12–23	1 or more dmfs surfaces	1 or more smooth dmf surfaces.
24–35	1 or more dmfs surfaces	1 or more smooth dmf surfaces.
36–47	1 or more dmfs surfaces	1 or more cavitated, filled, or missing (due to caries) smooth surfaces in primary maxillary anterior teeth or dmfs score >4.
48–59	1 or more dmfs surfaces	1 or more cavitated, filled, or missing (due to caries) smooth surfaces in primary maxillary anterior teeth or dmfs score >5.

Another classification based on the stage of development of the dentition and severity of dental caries (initial and cavitated) was proposed by Veerkamp and Weerheijm (74). This system assumes that dental caries occurs in successive stages starting late in the first year (10 months) and ending in the fourth year of life (48 months). The four stages were referred to as: initial, damaged, deep lesions, and traumatic. During each stage, a different group of teeth are involved, and dental caries can range from enamel demineralization (opaque white demineralization) to cavitation involving enamel and dentine.

## Management

Maintaining primary dentition in a healthy condition is important for the well-being of the child. Primary dentition is required for proper mastication, esthetics, phonetics, space maintenance, and for prevention of aberrant habits. Reducing dental plaque formation, changing the bacterial composition of plaque, and modification of dietary habits are essential for the prevention of dental caries. Prevention of the progress of the ECC can be achieved with the aid of restorations, diet counseling, educating parents regarding decay promoting feeding behaviors, maintain good oral hygiene, and the use of preventive agents like topical fluorides (75). The management of ECC is expensive, often requiring extensive restorative treatment and extraction of teeth at an early age. General anesthesia or deep sedation may be required at times, since young children lack the ability to cope with the extensive treatment procedures (76). Chemotherapeutic agents, such as povidone-iodine and chlorhexidine, have shown antimicrobial effect against the most cariogenic SM (77, 78). Chlorhexidine varnish is applied to protect the tooth surface (79).

In addition, fluorides are very effective in preventing dental caries, including fluoride toothpaste, water fluoridation, fluoride mouth rinse, and professional topical fluoride application, primarily by inhibiting mineral loss from the tooth. Tooth paste containing fluoride showed a strong preventive effect in young permanent teeth (80). Professionally applied fluoride varnishes and supervised use of fluoride mouth rinses also showed reduction in childhood caries (81, 82). The application of casein phosphor peptide (CPP) could stabilize the calcium and phosphate thereby preserving them in an amorphous or soluble form known as amorphous calcium phosphate (ACP). Calcium and phosphate are essential components of enamel and dentine and form highly insoluble complexes in the presence of CPP. CPP–ACP complexes can prevent tooth demineralization and improve enamel remineralization and enhance fluoride activity. Hence, the application of CPP–ACP-based compounds helps in the prevention of dental caries (83, 84).

### Preventive Measures

**Figure 2:**
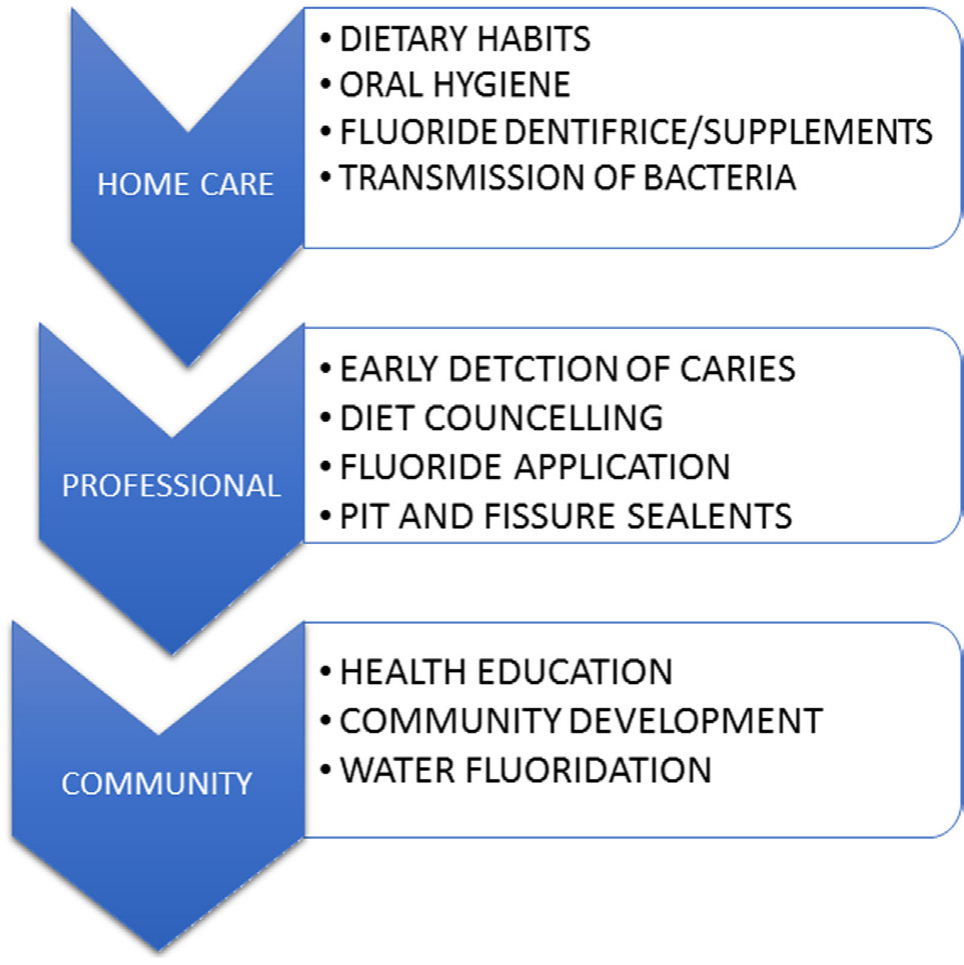
Strategies for the prevention of early childhood caries at various levels.

Early childhood caries preventive strategies should begin with prenatal education of expectant parent(s), progress through the perinatal period, and continue with the mother and infant (Figure 2). Adequate dental treatment and oral hygiene measures during pregnancy can reduce or delay ECC in infants (85). Parents also should be advised to maintain optimal dental health during pre- and postnatal periods (86, 87). Measures should be taken in educating parent/caregiver about the etiology and prevention of ECC (88). Nurses are also in a position to carry out prevention efforts for infants, toddlers, and their families and can provide counseling and support for children who suffer from ECC (86). A recently published multilevel conceptual model, incorporating influences of ECC exerted at the individual, family, and community level suggests that both social and behavioral change is important in the prevention of this oral disease (89). Use of probiotics chewable tables or supplements also showed some evidence in controlling the caries in children. However, its effectiveness to prevent ECC is still under investigation (90, 91).

## Conclusion

ECC is a chronic, infectious disease affecting young children, and constitutes a serious public health problem. It is one of the most common preventable diseases and is on the rise worldwide. ECC is a multifactorial disease consequent to the interaction of cariogenic microorganisms, exposure to carbohydrates, inappropriate feeding practices, and a range of social variables. It can affect a child’s well-being, learning ability, and quality of life. This virulent form of dental caries begins soon after dental eruption mainly on the smooth surfaces of the teeth, which progress at a rapid state. It has a lasting detrimental impact on the dentition. The associated pain from dental caries has a negative impact on children’s emotional status, sleep patterns, and ability to learn or perform their usual activities. A wide range of risk factors are associated with ECC in children from underprivileged and low socioeconomic status. Oral health has been recognized as an essential component of general health and quality of life. Hence both oral disease prevention and oral health promotion should be included as an integral part of chronic disease prevention and general health promotion programs.

## Author Contributions

SA and PA contributed in the concept, design of the review drafting, and revising the manuscript.

## Conflict of Interest Statement

The authors declare that the research was conducted in the absence of any commercial or financial relationships that could be construed as a potential conflict of interest.
